# Multimodal Biomarker Characterization of the ALS/FTD Spectrum: A Real-World Clinical Dataset Analysis

**DOI:** 10.3390/ijms262311496

**Published:** 2025-11-27

**Authors:** Sasha Mukhija, Lisa Hering, Simon J. Schreiner, Franz Lehner, Jan Loosli, Claudio Togni, Ferdinand Otto, Mario Ziegler, Tobias Weiss, Hans H. Jung, Nils Briel

**Affiliations:** 1 Department of Neurology, University Hospital of Zurich, 8091 Zurich, Switzerland; sasha.mukhija@usz.ch (S.M.);; 2 Neuroscience Center Zurich, University of Zurich, 8057 Zurich, Switzerland; 3 Faculty of Medicine, University of Zurich, 8032 Zurich, Switzerland

**Keywords:** pTau:tTau ratio, amyotrophic lateral sclerosis, frontotemporal dementia, Alzheimer’s disease, cerebrospinal fluid, diagnostic biomarkers

## Abstract

Diagnosis and prognosis of the amyotrophic lateral sclerosis and frontotemporal dementia (ALS/FTD) spectrum remain largely dependent on clinical assessments due to a lack of established fluid biomarkers. While neurofilaments and the cerebrospinal fluid (CSF) phosphorylated-tau/total-tau ratio (pTau:tTau) have been studied, their limitations, including their lack of clinical implementation and low specificity, necessitate multimodal approaches. This study aimed to characterize the biological features of the ALS/FTD spectrum through integration of clinically available parameters. We conducted a retrospective, single-center, cross-sectional study analyzing routinely collected clinical, neuroimaging, CSF, and serum data from 229 samples, including 45 from patients with ALS, 26 from patients with FTD, 158 from patients with other neurodegenerative diseases, and 29 from cognitively healthy controls. We implemented propensity score-weighted comparisons, an F1 score-based optimal cut-point determination for the pTau:tTau ratio, and a regularized XGBoost-based multimodal feature modeling approach. The biomarker and model performance was evaluated by the area under the precision–recall curve (AUC-PR). Feature importance analysis identified characteristic indicators of the ALS/FTD spectrum. Consistent with the prior literature, the pTau:tTau ratio was significantly reduced in ALS/FTD, but the classification performance was modest (AUC-PR 0.32). A multimodal model integrating clinical, biofluid, and neuroimaging features achieved a notably better performance (AUC-PR 0.75). Feature importance analysis revealed an ALS/FTD signature beyond the pTau:tTau ratio characterized by higher global cognition, younger age, an altered Aβ42/pTau ratio, and immunoglobulin changes (CSF IgG:IgA, serum IgG). Integration of clinical routine data centered on tau, amyloid, and immunological pathophysiology as well as temporal disease dynamics provide a contextualized biological characterization of the ALS/FTD spectrum. This approach offers a foundation for hypothesis generation regarding ALS/FTD pathophysiology and biomarker-supported diagnosis.

## 1. Introduction

Amyotrophic lateral sclerosis (ALS) is a rare and fatal neurodegenerative disease characterized by progressive muscle weakness and atrophy, due to the degeneration of both upper and lower motor neurons [1,2]. The median survival from motor manifestation is approximately three years [3]. Diagnosing ALS can be particularly challenging due to the absence of definite biomarkers. Instead, the diagnosis typically relies on a combination of extensive clinical and ancillary tests aimed at assessing motor function and ruling out mimicking conditions [4,5]. Therefore, accurate and early diagnosis is critical, as it allows for the timely initiation of supporting measures and potential disease-modifying therapies in selected cases [6].

Clinically manifest frontotemporal dementia (FTD), meeting established diagnostic criteria for dementia, develops in 10–15% of individuals with ALS, whereas an additional ~30–40% exhibit milder cognitive and/or behavioral changes within the ALS–frontotemporal spectrum that do not reach the dementia threshold [7,8]. FTD itself presents with variable phenotypes with progressive changes in behavior, executive function, and language abilities. FTD exhibits characteristic frontotemporal lobar degeneration and is considered the second most common form of dementia in people under the age of 65 [9]. It shares important clinical, genetic, and pathological properties with ALS [7]. Between 10 and 50% of FTD patients develop ALS-typical features throughout the disease course [10,11,12]. Furthermore, 95% of ALS cases and 50% of FTD cases exhibit TAR DNA-binding protein 43 (TDP-43) proteinopathy, albeit with varying neuropathological patterns. Given this clinical overlap and shared biology, both entities are considered to be on an ALS/FTD spectrum—the entirety of which we refer to hereunder as ALS/FTD [13].

The prognosis of ALS/FTD can vary widely among individuals, influenced by factors such as specific genetic mutations and the phenotypic manifestation of the disease. Pleiotropic effects of genetic mutations may lead to diverse clinical presentations, even within the same family. This variability underscores the need to develop prognostic models that incorporate clinical, phenotypic, and biological factors to better predict outcomes for patients. Despite ongoing research, there remains a critical need for reliable biomarkers for ALS/FTD, which complicates both diagnosis and treatment [13].

The phosphorylated-tau/total-tau (pTau:tTau) ratio in cerebrospinal fluid (CSF) is markedly reduced in ALS/FTD spectrum disorders, including TDP-43 proteinopathies, compared to other neurodegenerative diseases, with intermediate reductions in 4R tauopathies like progressive supranuclear palsy and corticobasal degeneration. This reduction is primarily driven by a disproportionate increase in tTau in relation to unchanged or only mildly reduced pTau levels in ALS, FTD, and tauopathies, resulting in low ratio values. The pTau:tTau ratio has shown robust diagnostic accuracy in distinguishing ALS from tauopathies and non-neurodegenerative controls [14,15,16].

Other investigations into canonical Alzheimer’s disease (AD) biomarkers (i.e., pTau-181, pTau-217, pTau-231) have noted significant increases in the blood of ALS patients as well—a finding that has been confirmed by independent groups and challenges the broader disease specificity of these markers [17,18]. However, blood-based phosphorylated-TDP-43-409/410 seems to be a promising candidate marker of TDP-43 proteinopathy [18,19]. Neurofilament light (NfL), a marker of neuroaxonal injury, has gained attention as a prognostic and stratifying biomarker rather than a diagnostic biomarker in ALS, FTD, and other rapidly progressive neurodegenerative diseases [20].

Studies aimed at understanding the molecular pathomechanisms of ALS and FTD have identified deregulation of CSF proteins associated with neurodegeneration, muscle atrophy, metabolic changes, synaptic and vesicle transport function [21], and neuroinflammation [22], highlighting potential pathways for candidate marker identification. While an increased CSF serum albumin ratio is associated with a faster progression in individuals with ALS [23], mechanistically, the loss of TDP-43 function in the endothelium might cause blood–brain barrier dysfunction [24]. Regarding adaptive immune responses, a selective intrathecal IgG1 and IgG3 synthesis has been noted without corresponding IgA production subclasses [23,25]. Furthermore, increased CD4+ and CD8+ T-cell activation profiles have been reported in ALS [26], and markers of glial activation can be detected in patients with ALS [27]. Eventually, the current diagnostic frameworks for ALS and FTD will advocate for the assessment of CSF basic and serological profiles to rule out autoimmune or infectious etiologies. Given this, inflammatory markers, including immunoglobulin profiles and measures of the blood–brain barrier integrity, may provide insights into disease mechanisms and offer potential differential diagnostic utility.

Most of these biomarkers require either further clinical validation, lack clinical routine application, or provide only limited information for differential diagnosis in ALS/FTD. While motor-predominant ALS presents comparably distinct symptoms, the broader ALS/FTD spectrum—particularly cognitive and behavioral presentations—poses significant diagnostic uncertainty. To elucidate differential biological insights distinctive for ALS/FTD against other neurodegenerative diseases, we conducted a systematic comparison of their CSF, serological, clinical, and neuroimaging biomarker profiles.

## 2. Results

### 2.1. Cohort Characteristics

A total of 229 samples from 214 patients were included in the analysis, distributed across seven diagnostic labels: ALS (*n* = 45); FTD (*n* = 26); 4R_Tau (*n* = 34); AD (*n* = 52); NPH (*n* = 45); LBD (*n* = 18); and controls with normal cognitive aging (NCA) (*n* = 29). Detailed demographic and clinical features are presented in Table 1.

Age at estimated symptom onset differed significantly between groups (Kruskal–Wallis, *p* < 0.001), ranging from a mean (SD) of 60.0 (13) years in the NCA group to 75 (7) years in NPH. Similarly, age at first LP varied (overall *p* < 0.001), being lowest in NCA (61 [12] years) and highest in NPH (77 [6] years). The interval between estimated symptom onset and LP was the shortest in NCA (1.1 [1] years) and the longest in LBD (4.4 [9] years, *p* < 0.05).

The sex distribution also varied across diagnoses (χ^2^/Fisher, *p* < 0.05), with the highest proportion of male participants observed in FTD (85%) and the lowest in AD (51%). The prevalence of comorbid conditions, such as neoplastic disease, also differed nominally between groups, being most frequent in LBD (44%) and 4R_Tau (36%, statistical comparison omitted given the low occurrence rate). Diabetes mellitus, CKD, arterial hypertension (*p* = 0.10), and autoimmune comorbidities did not differ between diagnostic labels.

For subsequent analyses, these seven diagnostic labels were collapsed into four major diagnostic groups (ALS/FTD; 4R_Tau; DC [disease controls: AD, LBD, and NPH]; and NCA) that share inherent intra-group biological–clinical properties or serve as disease or normal control populations (cohort characteristics provided in Supplementary Table S1). To assess whether age at LP or sex influences biomarker levels, we conducted linear regressions with interaction terms with ALS/FTD spectrum diagnosis. Only the κFLC index showed significant age-dependent effects (ß = +0.973 ± 0.268, adjusted *p*-value = 0.042), while all other biomarkers demonstrated consistent diagnostic performances across demographic strata (Supplementary Table S2).

### 2.2. CSF Biomarker Profiles Across Neurodegenerative Diseases

Given the established diagnostic challenges in differentiating ALS/FTD from other neurodegenerative diseases, we systematically explored multimodal biomarker signatures to characterize distinct proteinopathies and evaluate their differential diagnostic potentials.

**Figure 1:**
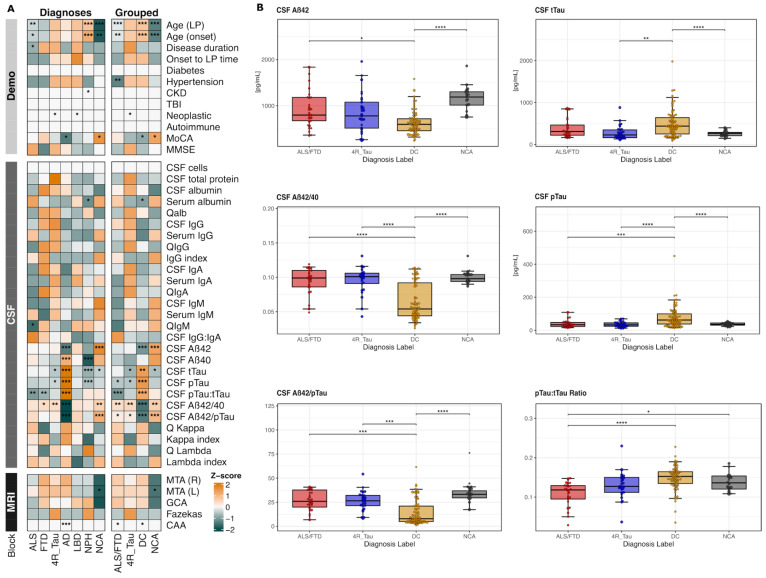
Exploratory and confirmatory analyses of multimodal markers across neurodegenerative diseases. (**A**) Heatmap of clinical, CSF, and neuroimaging variables across diagnoses and grouped. Color fill corresponds to variable-wise z-transformed values. Asterisks indicate one-vs.-all statistical comparisons using Wilcoxon tests; * *p* < 0.05, ** *p* < 0.01, *** *p* < 0.001. (**B**) Propensity score-weighted biomarker distributions across four diagnostic groups (ALS/FTD; 4R_Tau; disease controls; NCA). Box-and-jitter plots show the six CSF biomarkers with significant overall group differences after inverse probability weighting (IPW). Weighted medians and interquartile ranges are depicted. Asterisks indicate pairwise statistical comparisons using Wilcoxon tests; * *p* < 0.05, ** *p* < 0.01, *** *p* < 0.001, **** *p* ≤ 0.0001. Abbreviations: CAA: cerebral amyloid angiopathy; CKD: chronic kidney disease; Demo: demographic features; GCA: global cortical atrophy; MMSE: Mini-Mental Status Examination; MoCA: Montreal Cognitive Assessment; MTA (L/R): mesiotemporal atrophy; Qalb: CSF–serum albumin ratio; QIgG/QIgA/QIgM: CSF–serum immunoglobulin quotient; TBI: traumatic brain injury.

Exploratory analyses revealed distinctive biomarker, clinical, and demographic feature patterns across the seven diagnosis labels, as visualized in the comprehensive heatmap (Figure 1A). Within this heatmap, the individual ALS and FTD groups exhibited similar biomarker profiles (left panel), supporting their grouping as a unified ALS/FTD spectrum for subsequent analyses (further supported in Supplementary Figure S2). The heatmap also demonstrated clear separation between grouped diagnoses, with ALS/FTD showing notably distinct values compared to the remaining groups, especially within the recognized CSF parameters. As expected, AD exhibited not only elevated tau-related markers and reduced Aβ42 but also low immunoglobulin isotype levels, while both ALS and FTD displayed an inverse pattern with intermediate tTau increases and unchanged amyloid concentrations but heterogenous shifts in immunoglobulin isotypes. The 4R_Tau profile partly resembled the ALS/FTD pattern, though with lower tTau levels and differential immunoglobulin-related alterations. LBD cases had comparably neutral Aβ- and tau-related markers, while NPH cases depicted the strongest Aβ40, pTau, and tTau reductions. The NCA feature profile showed the physiological states of the cognitive performance, biomarker levels, and neuroimaging patterns.

To account for differences in the demographic and clinical covariates, we applied inverse probability weighting (IPW) and repeated the analysis on the four collapsed diagnostic groups: ALS/FTD, 4R_Tau, DC, and NCA. Of the 17 CSF biomarkers examined, six reached statistical significance after IPW adjustment: Aβ42, Aβ42/Aβ40, tTau, pTau, and the pTau:tTau and Aβ42/pTau ratios (Figure 1B, Supplementary Figure S3). ALS/FTD demonstrated a distinct neurochemical signature characterized by significantly higher Aβ42 compared to DC. The PTau levels and pTau:tTau ratio were markedly lower in ALS/FTD relative to DC. Both principal markers of AD pathophysiology, the Aβ42/Aβ40 and Aβ42/pTau ratios, were significantly lower in DC compared to ALS/FTD.

These findings validate and expand a distinct neurochemical fingerprint for the ALS/FTD spectrum that reflects the underlying non-Alzheimer pathophysiology and provides a foundation for biomarker-based differential diagnosis. Importantly, these AD vs. non-AD contrasts do not diminish the discriminatory value of markers relevant to ALS/FTD. Rather, they provide a biological reference against which more subtle non-AD neurodegenerative patterns, including ALS/FTD, can be contextualized.

### 2.3. Diagnostic Value and Limitations of the pTau:tTau Ratio

The pTau:tTau ratio as a diagnostic marker of ALS/FTD reflects non-Alzheimer pathophysiology with a disproportional tTau release from neuroaxonal–synaptic damage and only modestly changed pTau levels. We next assessed its differential diagnostic value and limitations across the studied disease spectrum.

**Figure 2:**
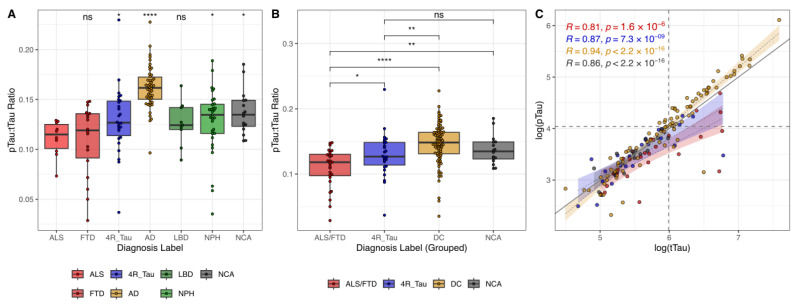
The pTau:tTau ratio as an indicator of ALS/FTD. (**A**) Between-group comparisons of pTau:tTau ratio between ALS and remaining diagnoses were assessed using Wilcoxon tests. * *p* < 0.05; **** *p* < 0.001; ns—not significant. (**B**) Between-group differences in pTau:tTau ratio across grouped diagnoses were assessed using Wilcoxon test. * *p* < 0.05; ** *p* < 0.01; **** *p* < 0.001; ns—not significant. (**C**) Scatterplot of the relationship between pTau and tTau. Both axes are scaled logarithmically. Dashed vertical and horizontal lines indicate tTau and pTau reference cutoffs, respectively. Linear regression lines with SD indicate different slopes for each diagnosis group.

We could replicate distinct distributional patterns of the pTau:tTau ratio across neurodegenerative diseases. The ALS and FTD groups consistently demonstrated the lowest median ratio values, while the AD group exhibited the highest (Figure 2A,B). The 4R_Tau, LBD, NPH, and NCA groups showed intermediate values, with the 4R_Tau group’s median appearing slightly lower than the remaining ones. The ability to predict ALS/FTD vs. non-ALS/FTD cases based only on the pTau:tTau ratio (optimal cut-point = 0.120) was modest (sensitivity = 0.61; specificity = 0.80; AUC-PR = 0.32; AUC = 0.78; Supplementary Table S3).

When examining the correlational patterns of the pTau and tTau CSF levels, AD samples exhibited a high level of correlation (Figure 2C). As expected, AD cases consistently presented with both elevated pTau and tTau values, aligning in the upper-right quadrant. Conversely, ALS/FTD samples with lower values for both parameters congregated in the lower-left quadrant. Both ALS/FTD and 4R_Tau showed less steep regression slopes, emphasizing the distinct tTau-to-pTau decoupling pattern. Notably, these groups displayed increased scatter at lower concentrations of both tTau and pTau. The greater variability at low concentrations was consistent across all diagnostic groups, possibly attributable to measurement inaccuracy at the assays’ lower limit of detection.

We further investigated the relationship between the pTau:tTau ratio, neuroimaging features, and basic CSF parameters. The 4R_Tau group showed a significant positive correlation between the pTau:tTau ratio and the follow-up/survival time (R = 0.44, *p* = 0.0428) (Supplementary Figure S4).

In summary, the CSF pTau:tTau ratio exhibits group differences, aiding the diagnostic differentiation of ALS/FTD in the studied disease groups, but comes with technical limitations in low-concentration ranges and in the delineation of 4R_Tau.

### 2.4. A Multimodal Biomarker Panel for ALS/FTD Discrimination

To systematically characterize the biological signature of ALS/FTD and identify discriminatory patterns that distinguish this spectrum from other neurodegenerative diseases with partly overlapping clinical presentations but distinct presumed proteinopathies, we developed a regularized machine learning approach integrating routinely available features from clinical examination, CSF neurochemistry, and neuroimaging.

**Figure 3:**
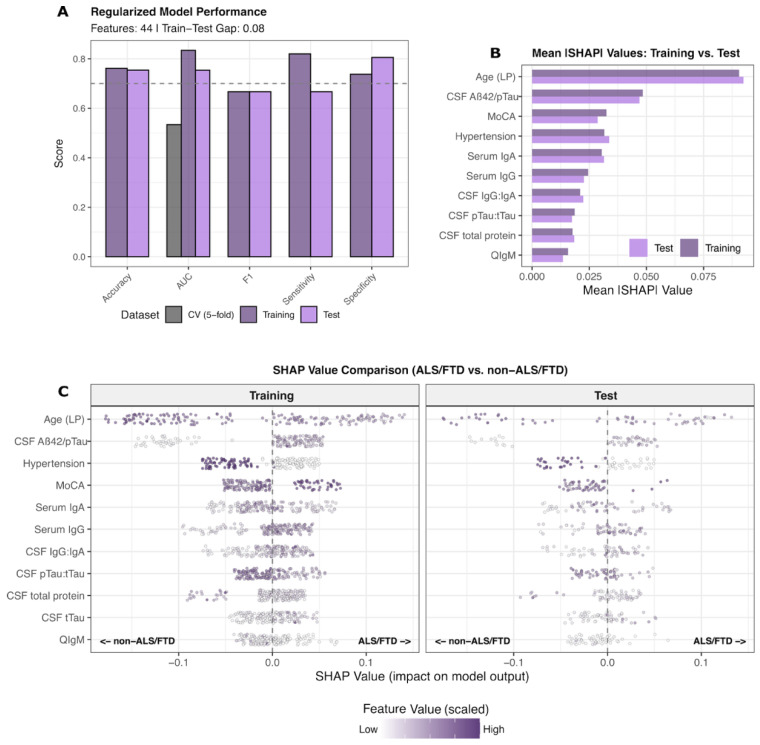
Multimodal models for ALS/FTD characterization. (**A**) Performance metrics across cross-validation (5-fold), training, and test datasets (45 features, train–test gap in AUC: 0.15). (**B**) Comparison of SHAP values in top 10 features by mean value in training and test sets. Similar SHAP values indicate consistent feature importance. (**C**) SHAP value distribution for training and test sets. Points represent individual samples, colored by scaled feature values (white—low; purple—high). Positive SHAP values indicate contributions toward ALS/FTD classification, and negative SHAP values indicate contributions against ALS/FTD classification; negative values indicate contributions toward non-ALS/FTD. Abbreviations: AUC: area under the curve; MoCA: Montreal Cognitive Assessment; SHAP: SHapley Additive exPlanations.

A single regularized multimodal XGBoost model incorporating 44 features was trained on a 75% split of the cohort to discriminate ALS/FTD (*n* = 71) from non-ALS/FTD entities (4R_Tau, AD, LBD, NPH, and cognitively normal controls; *n* = 158), with the final performance evaluated in the remaining unseen 25% test set cases (Figure 3A, Supplementary Table S4). The model achieved training and test AUC-PR values of 0.83 and 0.75, respectively. Performance metrics, including accuracy, sensitivity, and specificity, showed consistent discrimination capacity across train–test splits (Figure 3A).

SHAP-based feature contribution analysis revealed a distinct predictive pattern in ALS/FTD (Figure 3B,C). Top-ranked features included lower age at LP, the CSF pTau:tTau ratio, serum IgA, and frequency of arterial hypertension, on the one hand. On the other hand, higher CSF Aβ42/pTau and CSF IgG:IgA ratios, serum IgG levels, and MoCA scores were predictive of ALS/FTD. The SHAP value distributions demonstrated the concordant directionality of biological contributions between training and test partitions, with mean absolute SHAP comparisons confirming stable importance rankings across independent datasets. Although, in this regularized model, the pTau:tTau ratio contributed relevant information to the prediction task, it was superseded by serological, AD, and clinical features.

The nominal increase in the CSF IgG:IgA ratio in ALS, FTD, and, to a lesser extent, 4R_Tau, compared to the remaining groups (Figure 1A, exploratory), was not significant upon more detailed analysis (Supplementary Figure S5). However, the CSF IgG:IgA ratio was significantly negatively correlated with the Qalb in ALS/FTD.

These findings establish a distinct biological profile for ALS/FTD characterized by altered tTau dynamics, absent amyloid metabolism, immunoglobulin dysregulation, relatively preserved global cognitive function, and younger age at presentation—distinguishing this spectrum from AD-type neurodegeneration, primary tauopathies, and other proteinopathies.

## 3. Discussion

This study reveals a distinctive pathophysiological profile for the ALS/FTD spectrum, characterized by a reduced pTau:tTau ratio, altered amyloid and immunoglobulin dynamics, as well as relatively preserved cognition and younger age—patterns that distinguish ALS/FTD from AD-type neurodegeneration, primary tauopathies, and other neurodegenerative entities. Our multimodal approach provides a contextual view into differential pathomechanisms across neurodegenerative diseases, with contact points for potential diagnostic and therapeutic applications.

A major practical consequence of persistent diagnostic uncertainty in ALS and FTD is that many treatment decisions, supportive interventions, and referrals are routinely deferred until the clinical picture and ancillary testing results evolve sufficiently to confirm the diagnosis. This approach is common in the real world but can inadvertently prolong periods during which patients are left without appropriate multidisciplinary care, prognostic counseling, or access to disease-modifying trials [28]. A validated biomarker panel tested for at an early stage that reliably reduces diagnostic uncertainty would therefore have direct clinical impact by shortening time-to-diagnosis and mitigating potentially harmful delays in initiating patient-centered management.

Although CSF biomarkers differed most significantly when comparing AD against the remaining diagnoses, this reflects the well-established and highly specific neurochemical signature of AD rather than a limitation in ALS/FTD discrimination. By contrast, our multimodal models, which integrate CSF, clinical, and neuroimaging features, demonstrated good discriminatory performances for ALS/FTD despite the more modest univariate group differences. Thus, the AD-driven separation serves primarily as a biological benchmark rather than the focus of diagnostic differentiation.

Our findings corroborate and extend previous observations regarding the distinctive neurochemical signature of ALS/FTD. The consistently reduced pTau:tTau ratio in ALS/FTD reflects the underlying non-Alzheimer pathophysiology, primarily driven by disproportionate tTau release from neuroaxonal and synaptic damage with relatively preserved or mildly reduced pTau levels [14,15,16,29]. This pattern aligns with recent meta-analytic evidence validating consistently decreased pTau:tTau ratio values in ALS patients compared to controls (SMD: −0.84 [−1.16 to −0.53]) [30]. Critically, the pTau:tTau ratio demonstrates modest discriminatory capacity when used in isolation, is influenced by a higher spread at low concentration ranges, and, in turn, has limited diagnostic utility at the individual-subject level. While CSF pTau in ALS is also lower than that in healthy controls [31], recent evidence has demonstrated that phospho-tau species (pTau181, pTau217) are elevated in the sera of ALS patients as well, challenging the disease specificity of these canonical AD markers, if applied without clinical context [17]. Our CSF-based approach provides higher pathophysiological specificity by directly reflecting CNS processes, yet the heterogeneous pathological substrate underlying FTD variants (including TDP-43, tau, and other proteinopathies) limits the utility of tau-based markers alone. This underlines the need for sophisticated multi-parameter approaches or for TDP-43- and tau isoform-specific biomarkers to enable comprehensive FTD spectrum characterization [32].

When integrated with complementary clinical, serological, and neuroimaging features through machine learning, the biological profile of ALS/FTD was notably more distinctive, revealing patterns not captured by individual biomarkers. The prominence of preserved MoCA scores as a discriminatory feature in ALS and some FTD variants might reflect the relatively preserved cognitive function in pure motor presentations, supporting clinical phenotyping approaches that emphasize cognitive–motor divergence. The Edinburgh Cognitive and Behavioural ALS Screen (ECAS) has been specifically designed and validated to detect frontotemporal and executive dysfunction in ALS and may have provided a more nuanced cognitive profile of our ALS cohort [33].

The CSF IgG:IgA ratio emerged from the XGBoost modeling and SHAP analysis as a potentially distinctive marker, though its biological basis remains incompletely understood. Several pathophysiological mechanisms may account for the elevated CSF IgG:IgA ratio observed in ALS/FTD. Blood–brain barrier dysfunction, documented in approximately 30% of ALS patients by an elevated Qalb, facilitates the preferential CSF entry of serum-derived IgA over IgG. In genetic ALS/FTD, endothelial TDP-43 dysfunction appears to drive barrier breakdown, with subsequent immunoglobulin accumulation [24]. Consistent with this hypothesis, the CSF IgG:IgA ratio correlated with the Qalb in our ALS/FTD subgroup, suggesting that increased intrathecal IgA levels may reflect passive diffusion rather than local synthesis. Previous reports of a selective intrathecal synthesis of IgG1 and IgG3 without corresponding IgA production subclasses [23,25], alongside increased CD4+ and CD8+ T-cell activation in ALS [26], indicate adaptive immune involvement distinct from classical neuroinflammatory disorders. However, we did not replicate significant group-level differences in the intrathecal fractions of IgG and IgA in our cohort. Given these incongruous observations and the exploratory nature of our analysis, the IgG:IgA ratio should be interpreted as a descriptive composite marker whose relationship to barrier dysfunction versus disease-specific immune activation requires systematic investigation in independent cohorts.

Structural neuroimaging features contributed minimally to the ALS/FTD spectrum characterization in our unified modeling approach. This limited contribution likely reflects phenotypic heterogeneity within the spectrum and the absence of spinal imaging, which would identify cervical spinal disorders as a treatable ALS-mimic. While *cerebral* MRI remains valuable for excluding alternative pathologies and assessing atrophy patterns in FTD, our findings deprioritize the value of the included—and admittedly high-level—features for ALS/FTD diagnosis.

Future validation studies should incorporate blood-based biomarkers like NfL, emerging cryptic exon-based approaches, and extracellular vesicle TDP-43 detection that demonstrate high sensitivity and specificity for TDP-43 proteinopathies [34,35]. Observing longitudinal biomarker trajectories will be essential for capturing disease progression patterns and facilitating early detection in presymptomatic stages.

### Limitations

Several limitations warrant consideration. The single-center design may limit generalizability, and the modest sample sizes for certain diagnostic categories (LBD, 4R tauopathies) restrict statistical power for disease discrimination. This is of importance especially for machine learning approaches, where larger datasets are essential for validating our findings and expanding their generalizability to external cohorts. We mitigated this by implementing a balanced prediction and test set generalization approach and accounting for imbalanced class labels.

The use of clinical diagnostic criteria rather than autopsy confirmation in this study may have introduced misclassification biases, as disconcordance of clinical and neuropathological autopsy is documented [36]. We emphasize this as a common constraint of real-world, retrospective research, though we excluded cases with ambiguous diagnoses and applied established clinical diagnostic frameworks. Moving forward, it will be critical to validate these findings within autopsy-confirmed and/or genetic ALS/FTD cohorts to address this limitation and improve the classification reliability.

A further limitation concerns the use of the MoCA instead of an ALS-specific cognitive assessment, such as the ECAS [33]. However, the MoCA was the only uniformly available test across all diagnostic groups and thus permitted valid cross-disease comparisons. This may have led to underestimation of subtle ALS-specific cognitive and behavioral impairments.

Furthermore, although our study identified a pathophysiological profile contextualizing the pTau:tTau ratio, the absence of NfL, or other blood-based markers (e.g., serum NfL or TDP-43 derivatives), were unavoidable limitations tied to the clinical reliance on and availability of routine biomarkers. Eventually the studied disease spectrum did not account for typical motor-dominant ALS-mimics, including inflammatory, metabolic, and genetic entities, which warrants multicenter studies of more diverse patient cohorts.

## 4. Methods

### 4.1. Study Population and Study Design

In this retrospective study, we analyzed data from patients who underwent comprehensive clinical evaluation, CSF biomarker testing, neuroimaging, and additional ancillary testing as part of the routine work-up for suspected neurodegenerative or other neurological disorders. A total of 272 CSF neurochemistry records from patients diagnosed with neurodegenerative and non-neurodegenerative diseases were screened from the University Hospital Zürich (USZ) database, acquired from January 2016 to May 2025. After excluding those with undetermined diagnoses and acute-inflammatory CSF profiles (i.e., CSF cell count > 5/µL), 229 samples from 214 patients were retained.

We selected patients with diagnoses based on established clinical criteria [4,37,38,39,40,41,42,43], including ALS (Awaji-Shima criteria [4] were preferred over Gold Coast criteria given their higher specificity [37]), FTD (behavioral variant, primary progressive aphasia, motor neuron disease [38]), 4R tauopathy (probable 4R_Tau [39]), AD (revised ATN criteria) [40], presumed Lewy body diseases (LBD, i.e., Parkinson’s disease [dementia] [41] and dementia with Lewy bodies [42]), and idiopathic normal-pressure hydrocephalus (NPH) [43]. As controls, we included patients with non-neurodegenerative diagnoses (i.e., psychiatric and primary headache disorders) lacking any objective cognitive decline and over the age of 45, grouped as normal cognitive aging (NCA). Importantly, the included comparator groups (AD, 4R-Tau, LBD, NPH, NCA) do not represent classic ALS mimics and do not typically present with progressive muscle weakness or electrophysiological evidence of motor neuron pathology. Instead, these groups reflect the real-world population undergoing CSF biomarker evaluation in a tertiary care setting for cognitive and neurodegenerative diseases.

From selected patients, the following data were retrieved: diagnosis; sex; age at onset of symptoms; age at lumbar puncture (LP); follow-up time after LP; CSF biomarkers and relevant comorbidities, such as diabetes mellitus, arterial hypertension, chronic kidney disease (CKD), neoplasms, traumatic brain injury (TBI), and autoimmune disorders. Furthermore, magnetic resonance imaging (MRI) data, such as medial temporal lobe atrophy (MTA, right and left) scores, global cortical atrophy (GCA) scores, and Fazekas white matter hyperintensity grades were included. The clinical disease severity was assessed using the revised ALS Functional Rating Scale (ALSFRS-r), Montreal Cognitive Assessment (MoCA), Mini-Mental State Examination (MMSE), and Unified Parkinson Disease Rating Scale (UPDRS), with the genetic mutation status recorded, if available. Deceased patients had a registered death date, whereas the remaining ones were categorized as loss of follow-up until the last contact.

### 4.2. CSF Processing

LP was performed at the L3/4 or L4/5 intervertebral spaces using a sterile syringe. CSF neurochemistry analysis followed previously described protocols [44]. Briefly, the CSF white blood cell count was conducted using a Fuchs–Rosenthal chamber, within 1–2 h after LP. Cytologic differentiation was conducted if the CSF cell count exceeded 4/µL. Concentrations of albumin, immunoglobulins (IgG/A/M), and κ/λ-free light chains (κFLC, λFLC) were quantified in both CSF and serum by immune nephelometry. From these, CSF–serum ratios were calculated. The blood–CSF barrier integrity was assessed via the CSF–serum albumin ratio (Qalb). The intrathecal synthesis of immunoglobulins was evaluated through the application of Reiber’s formula [45] (for IgG/M/A, κFLC) and isoelectric focusing with immunoblotting (oligoclonal bands).

Canonical AD biomarkers, including amyloid β_1–42_ (Aβ42), amyloid β_1–40_ (Aβ40), the Aβ42/Aβ40 ratio, phospho-tau181 (pTau), and total-tau (tTau), were measured in CSF using the Fujirebio Lumipulse G600II system. The cutoff values were defined as follows: Aβ42: 500 pg/mL; Aβ42/Aβ40: 0.0069; pTau: 56.4 pg/mL, and the age-adjusted values for tTau were as follows: 300 pg/mL for patients ≤50 years; 450 pg/mL for those 51–70 years; and 500 pg/mL for patients >70 years.

### 4.3. Statistical Analysis

All statistical analyses were performed in RStudio with R 4.3.3 using the data.table, tidyverse, cobalt, nnet, survey, pROC, and caret packages and related visualization libraries. The source code used is available at https://github.com/nes-b/ALS-FTD-Classifier (accessed on 19 November 2025).

#### 4.3.1. Descriptive and Inferential Statistics

For each diagnostic group, we summarized the sample size, mean (±SD) age at estimated disease onset, age at LP, and time interval between disease onset and LP. The sex distribution and prevalence of comorbidities (diabetes mellitus, arterial hypertension, chronic kidney disease, neoplastic and autoimmune diseases) were expressed as percentages. Group comparisons were performed using Kruskal–Wallis tests for continuous variables and χ^2^ tests or Fisher’s exact tests (depending on the expected cell counts) for categorical variables. *P*-values were reported in a summary table.

Visualizations were generated using box-and-whisker plots with overlaid beeswarm plots for each biomarker, stratified by diagnosis group. Comparisons were first performed across all seven diagnostic groups and were then collapsed into four biologically motivated categories for secondary analyses: (i) ALS/FTD; (ii) 4R-Tau; (iii) DC (disease controls), comprising AD, LBD, and NPH; and (iv) NCA.

#### 4.3.2. Inverse Probability Weighting and Linear Regression Terms

To account for differences in the baseline characteristics between diagnostic groups, we applied inverse probability weighting (IPW) based on propensity scores. Propensity scores were estimated using logistic regression, including the covariates of arterial hypertension, CKD, diabetes mellitus, neoplastic disease, autoimmune disease, age at manifestation, and age at report. Each observation was then weighted by the inverse of the estimated probability of its group assignment [46,47].

Additionally, to assess whether age at LP or sex modify biomarker associations with ALS/FTD diagnosis, we conducted linear regression models with interaction terms (biomarker ~ ALS/FTD × age; and biomarker ~ ALS/FTD × sex). *P*-values were adjusted for multiple testing using the false discovery rate method. Spearman correlation analyses were performed to assess relationships among clinical, CSF, and neuroimaging variables, with correlation coefficients and *p*-values calculated using pairwise complete observations to handle missing data.

#### 4.3.3. Cut-Point Determination and Supervised Modeling

The best binary cut-point of the continuous pTau:tTau ratio values was determined by maximizing the F1-score, which balances the harmonic mean of precision and recall and accounts for class imbalances.

For supervised machine learning, the cohort was stratified into 75% training and 25% testing splits, balancing information to be learned and leaving a substantial remainder for testing the generalization ability in the training and testing sets, respectively.

Features were divided into three sets: clinic demographics (sex, age, and disease duration; cognition and motor function); CSF variables (i.e., CSF cytology, CSF chemistry, immunoglobulins and light chains, AD CSF biomarkers); and (semi-)quantitative MRI pathology patterns (i.e., MTA, GCA, Fazekas grade). Prior to modeling, feature correlations were assessed using Spearman correlation coefficients to identify multicollinearity patterns (Supplementary Figure S1). Missing data were handled using Multiple Imputation by Chained Equations [40], with m = 30 imputations using predictive mean matching and a maximum of 20 iterations. Test set imputation was performed using training set parameters to prevent data leakage.

Extreme Gradient Boosting (XGB) [48] was chosen for its robust performance, built-in strategies for mitigating data missingness, and intrinsic feature importance metrics. A comprehensive hyperparameter search was performed using 5-fold cross-validation including the maximum tree depth, learning rate, minimum child weight, subsample, colsample by tree, lambda, alpha, and gamma. The optimal hyperparameter configuration was selected using a composite score that balanced the cross-validation area under the precision–recall curve (AUC-PR), the standard deviation of the AUC-PR in cross-validation, and the train–test generalization gap: score = mean(AUC-PR_CV_) − 0.5 × sd(AUC-PR_CV_) − 0.4 × (Train-Test-Gap). This approach prioritized models with better generalization over those with higher training performances. To allow these models to capture gradually more complexity, the maximum depth was increased by +1. The cross-validation and final ALS/FTD models employed a binary-logistic objective to maximize the AUC-PR for classification of the positive class, thereby accounting for the underrepresented positive-class labels (i.e., ALS/FTD ~ 0.3).

#### 4.3.4. Feature Importance and SHAP Analysis

To interpret model predictions and identify the most influential biomarkers, we computed SHapley Additive exPlanations (SHAP) values for both training and test sets using the SHAPforxgboost R package (https://github.com/liuyanguu/SHAPforxgboost, accessed on 19 November 2025). SHAP values quantify each feature’s contribution to individual predictions by measuring the change in model output when that feature is included versus when it is excluded.

Feature values were scaled within each variable to enable comparison across different measurement scales (normalized to 0–1 range). The SHAP importance was visualized using point-jitter plots showing the distribution of SHAP values colored by scaled feature values. The mean absolute SHAP values were compared between training and test sets to assess the consistency of the feature importance across datasets. Feature rankings were evaluated using rank correlation and absolute rank differences to quantify the stability of the importance patterns.

## 5. Conclusions

This study demonstrates that a machine learning integration of routine clinical and biofluid data achieves biologically meaningful ALS/FTD classification, challenging single-marker paradigms and supporting emerging multimodal diagnostic frameworks for TDP-43-associated phenotypes. Beyond discrimination, the observed interplay between CSF tau dynamics and immunoglobulin profiles and clinical features generates testable hypotheses linking blood–brain barrier dysfunction and immune activation to ALS/FTD pathophysiology. Prospective studies in larger, autopsy- or genetically confirmed, and multicenter cohorts integrating blood-based and TDP-43-related biomarkers and assessing longitudinal trajectories from presymptomatic stages to ALS–ALS/FTD overlap will be essential to validate and extend this multimodal framework.

## Figures and Tables

**Table 1:** Cohort characteristics.

Variable	ALS	FTD	4R_Tau	AD	LBD	NPH	NCA	*p*-Value
n samples [cases]	45 [42]	26 [20]	31 [31]	47 [46]	16 [14]	40 [37]	24 [24]	
Age at onset, y (mean ± SD)	65 (11)	67 (7)	72 (7)	70 (10)	70 (10)	75 (7)	60 (13)	<0.001
Age at LP, y (mean ± SD)	67 (11)	69 (6)	74 (6)	72 (10)	75 (7)	77 (6)	61 (12)	<0.001
Onset → LP interval, y (mean ± SD)	1.3 (3)	2.5 (3)	2.5 (2)	1.8 (1)	4.4 (9)	1.8 (2)	1.1 (1)	<0.05
No. Male (%)	29 (64%)	22 (85%)	14 (45%)	24 (51%)	12 (75%)	24 (60%)	13 (54%)	<0.05
No. Diabetes mellitus (%)	2 (4%)	5 (19%)	3 (10%)	6 (13%)	2 (13%)	9 (23%)	3 (13%)	
No. Arterial hypertension (%)	13 (29%)	7 (27%)	16 (52%)	24 (51%)	10 (63%)	18 (45%)	12 (50%)	0.1
No. Chronic kidney disease (%)	1 (2%)	2 (8%)	0 (0%)	4 (9%)	1 (6%)	7 (18%)	1 (4%)	
No. Neoplastic disease (%)	5 (11%)	3 (12%)	11 (36%)	10 (21%)	7 (44%)	5 (13%)	2 (8%)	
No. Autoimmune disease (%)	0 (0%)	0 (0%)	0 (0%)	0 (0%)	0 (0%)	2 (5%)	0 (0%)	

Kruskal–Wallis and χ^2^/Fisher tests. *p*-values shown only for comparisons with ≥5 occurrences per group.

## Data Availability

The data presented in this study are available on request from the corresponding author due to privacy and ethical reasons.

## Supplementary Materials

The following supporting information can be downloaded at: https://www.mdpi.com/article/10.3390/ijms262311496/s1.
