# Supplementary material for: Multimodal Biomarker Characterization of the ALS/FTD Spectrum: A Real-World Clinical Dataset Analysis
Source: Int J Mol Sci. 2025 Nov 27;26(23):11496. doi: 10.3390/ijms262311496 (PMC12692265; doi:10.3390/ijms262311496)
Supplement: Supplementary file 1 [file ijms-26-11496-s001.zip › supplemental_data_rev1_clean.pdf]

Supplementary Data

Multimodal Biomarker Characterization of the ALS/FTD Spectrum: A Real-World Clinical Dataset Analysis

Sasha Mukhija[1,2], Lisa Hering[1,3], Simon J. Schreiner[1,2], Franz Lehner[1,2], Jan Loosli[1,2], Claudio Togni[1,2], Ferdinand Otto[1,2], Mario Ziegler[1,2], Tobias Weiss[1,2], Hans H. Jung*[1,2], Nils Briel*[1,2] (*contributed equally)

Author Affiliations

1. Department of Neurology, University Hospital of Zurich, Zurich, Switzerland
2. Neuroscience Center Zurich, University of Zurich, Zurich, Switzerland
3. Faculty of Medicine, University of Zurich, Zurich, Switzerland

Corresponding author: Nils Briel, MD, Department of Neurology, University Hospital of Zurich, Zurich, Switzerland, Frauenklinikstrasse 26, 8091 Zürich, Switzerland (Nils.Briel@usz.ch)

Supplementary Data

Variable	AD	4R_Tau	DC	NCA	p-value
n	71	31	103	24	
Age at onset, y (mean ± SD)	66 (10)	72 (7)	72 (9)	60 (13)	**<0.05**
Age at LP, y (mean ± SD)	67 (10)	74 (6)	74 (8)	61 (12)	**<0.05**
Onset → LP interval, y (mean ± SD)	2 (3)	3 (2)	2 (4)	1 (1)	**<0.05**
No. Male (%)	51 (72 %)	14 (45%)	60 (58%)	13 (54%)	0.06
No. Diabetes mellitus (%)	7 (10 %)	3 (10%)	17 (17%)	3 (13%)	0.62
No. Arterial hypertension (%)	20 (28 %)	16 (52%)	52 (51%)	12 (50%)	**<0.05**
No. Chronic kidney disease (%)	3 (4 %)	0 (0%)	12 (12%)	1 (4%)	
No. Neoplastic disease (%)	8 (11%)	11 (36 %)	22 (21 %)	2 (8%)	**<0.05**
No. Autoimmune disease (%)	0 (0 %)	0 (0 %)	2 (2 %)	0(0%)	

Supplementary Table 1. Cohort characteristics of the collapsed 4 groups.

Sample size (n) indicates samples per group. DC stands for "disease control" and comprises of LBD, NPH and AD. Kruskal-Wallis and χ^2/Fisher tests.

Model Interaction	Biomarker (outcome)	Estimate	Std Error	P Value	P Value (adjusted)
**ALS/FTD x age at LP**	kapndc_kappa_index	9.73E-01	2.68E-01	0.000974	0.0419
	kapqc_kappa_quotient	5.02E+00	1.60E+00	0.003664	0.078769
	gca	-5.48E-02	2.20E-02	0.014858	0.212966
	amyloid_angiopathy	-1.97E-02	9.88E-03	0.050717	0.545202
	amyloid_b_1_42	1.34E+01	7.95E+00	0.093906	0.807592
	albsc_albumin_im_serum	-1.02E-01	6.65E-02	0.128248	0.919107
	amyloid_b_1_40	1.17E+02	8.56E+01	0.175394	0.942743
	kaifc_kappa_if	1.17E+00	8.30E-01	0.168102	0.942743
	age_at_manifestation	-1.61E-02	4.88E-02	0.741413	0.99023

	dm	1.94E-03	5.05E-03	0.701263	0.99023
	aht	4.19E-03	7.16E-03	0.558925	0.99023
	ckd	-2.22E-03	3.79E-03	0.558008	0.99023
	tbi	-2.05E-04	3.05E-03	0.946389	0.99023
	neoplastic	3.55E-03	1.04E-02	0.733409	0.99023
	autoimmune	9.85E-03	8.12E-03	0.226634	0.99023
	moca_score_1	4.59E-02	2.93E-01	0.87556	0.99023
	mta_right	-2.47E-02	4.00E-02	0.539138	0.99023
	mta_left	-4.49E-02	4.07E-02	0.274638	0.99023
	fazekas_grade	-1.63E-02	1.71E-02	0.341597	0.99023
	tplc_totalprotein_im_liquor	-4.68E-01	3.60E+00	0.896719	0.99023
	alblc_albumin_im_liquor	-1.47E+00	2.19E+00	0.503908	0.99023
	albqc_albumin_quotient	-3.00E-02	5.15E-02	0.561408	0.99023
	igglc_ig_g_im_liquor	-6.00E-02	2.78E-01	0.82936	0.99023
	iggsc_ig_g_im_serum	-1.51E-02	3.41E-02	0.658565	0.99023
	iggqc_ig_g_quotient	-3.46E-04	2.82E-02	0.99023	0.99023
	iggndc_ig_g_index	3.10E-04	1.09E-03	0.775799	0.99023
	igalc_ig_a_im_liquor	1.86E-02	5.60E-02	0.740018	0.99023
	igasc_ig_a_im_serum	1.59E-02	1.82E-02	0.383522	0.99023
	igaqc_ig_a_quotient	2.70E-03	2.11E-02	0.898458	0.99023
	igmlc_ig_m_im_liquor	5.07E-03	1.45E-02	0.726256	0.99023
	igmsc_ig_m_im_serum	3.78E-03	9.33E-03	0.685617	0.99023
	igmqc_ig_m_quotient	-4.15E-03	1.29E-02	0.748058	0.99023
	zelllc_gesamtzellzahl	1.52E-02	1.41E-02	0.282348	0.99023
	total_tau	4.55E+00	6.48E+00	0.483366	0.99023
	phospho_tau	4.39E-01	1.20E+00	0.714866	0.99023
	quotient_ab1_42_ab1_40	-1.42E-05	6.06E-04	0.981374	0.99023
	q_abeta42_p_tau	5.93E-02	3.02E-01	0.844599	0.99023
	lamqc_lambda_quotient	-5.96E-01	7.40E-01	0.427911	0.99023
	lamndc_lambda_index	-1.96E-02	8.20E-02	0.812612	0.99023
	iggif_ig_g_if	9.04E-02	1.12E-01	0.422186	0.99023
	pTau_tTau_ratio	5.13E-05	7.06E-04	0.942241	0.99023
	csf_igg_iga_ratio	-3.49E-02	8.41E-02	0.678243	0.99023
	dis_dur	1.61E-02	4.88E-02	0.741413	0.99023
**ALS/FTD x sexM**	albsc_albumin_im_serum	4.17E+00	1.49E+00	0.005596	0.246245
	age_at_report	-4.71E+00	3.00E+00	0.118214	0.658933
	age_at_manifestation	-3.83E+00	3.13E+00	0.221741	0.658933
	aht	-1.71E-01	1.50E-01	0.258212	0.658933
	moca_score_1	6.82E+00	3.35E+00	0.043751	0.658933

	amyloid_angiopathy	-2.43E-01	2.16E-01	0.266334	0.658933
	igalc_ig_a_im_liquor	-1.39E+00	1.20E+00	0.247445	0.658933
	igasc_ig_a_im_serum	-5.11E-01	3.96E-01	0.198368	0.658933
	igmlc_ig_m_im_liquor	-3.60E-01	3.25E-01	0.269564	0.658933
	igmsc_ig_m_im_serum	3.94E-01	2.01E-01	0.051633	0.658933
	igmqc_ig_m_quotient	-3.50E-01	2.97E-01	0.24013	0.658933
	kapqc_kappa_quotient	6.71E+01	4.62E+01	0.156453	0.658933
	kapndc_kappa_index	9.54E+00	8.16E+00	0.251057	0.658933
	lamqc_lambda_quotient	2.82E+01	1.57E+01	0.082736	0.658933
	lamndc_lambda_index	3.16E+00	1.77E+00	0.085277	0.658933
	kaifc_kappa_if	2.60E+01	1.92E+01	0.184196	0.658933
	pTau_tTau_ratio	-1.57E-02	1.35E-02	0.247535	0.658933
	csf_igg_iga_ratio	2.58E+00	1.84E+00	0.162492	0.658933
	iggqc_ig_g_quotient	-6.32E-01	6.10E-01	0.301173	0.697452
	iggsc_ig_g_im_serum	7.11E-01	7.41E-01	0.33833	0.744326
	neoplastic	1.69E-01	2.18E-01	0.438944	0.806371
	autoimmune	1.44E-01	1.69E-01	0.393354	0.806371
	iggndc_ig_g_index	-1.64E-02	2.40E-02	0.494401	0.806371
	igaqc_ig_a_quotient	-3.13E-01	4.58E-01	0.494819	0.806371
	zelllc_gesamtzellzahl	-2.26E-01	2.96E-01	0.447056	0.806371
	phospho_tau	-1.63E+01	2.30E+01	0.479227	0.806371
	dis_dur	-7.38E-01	1.02E+00	0.470784	0.806371
	fazekas_grade	-1.76E-01	3.38E-01	0.602077	0.880681
	igglc_ig_g_im_liquor	-2.85E+00	5.99E+00	0.634457	0.880681
	amyloid_b_1_42	7.32E+01	1.56E+02	0.640495	0.880681
	q_abeta42_p_tau	3.23E+00	5.99E+00	0.590636	0.880681
	iggif_ig_g_if	-1.21E+00	2.45E+00	0.621886	0.880681
	tplc_totalprotein_im_liquor	-3.18E+01	7.43E+01	0.669582	0.892776
	dm	3.35E-02	1.05E-01	0.749092	0.896907
	tbi	2.40E-02	6.34E-02	0.705494	0.896907
	alblc_albumin_im_liquor	1.40E+01	4.48E+01	0.754217	0.896907
	albqc_albumin_quotient	-3.54E-01	1.06E+00	0.739286	0.896907
	mta_right	1.97E-01	6.90E-01	0.77578	0.898272
	ckd	-1.39E-02	7.84E-02	0.859868	0.954079
	total_tau	-2.10E+01	1.25E+02	0.867345	0.954079
	mta_left	1.76E-02	7.03E-01	0.980134	0.980134
	gca	-1.66E-02	4.50E-01	0.970612	0.980134
	amyloid_b_1_40	1.27E+02	1.66E+03	0.938877	0.980134
	quotient_ab1_42_ab1_40	7.12E-04	1.17E-02	0.95163	0.980134

Supplementary Table 2. Age and sex interaction analysis for biomarker performance.

Linear regression interaction terms testing whether age at LP or sex modifies biomarker associations with ALS/FTD diagnosis. Models: biomarker ~ ALS/FTD × age or biomarker ~ ALS/FTD × sex. P-values are FDR-adjusted for multiple testing. One age interaction reached significance (kappa index, p.adjusted = 0.042); no sex interactions were significant, indicating biomarker performance is consistent across demographic strata.

Metric	Optimal cutpoint	F1_score	accuracy	sensitivity	specificity	AUC-ROC	AUC-PR	prevalence
**Value**	0.120	0.479	0.763	0.607	0.797	0.785	0.320	0.179

Supplementary Table 3. Performance metrics of the pTau:tTau ratio for ALS/FTD spectrum classification.

The optimal cutpoint was determined by maximizing the F1 score on 156 complete cases (ALS/FTD n = 28, non-ALS/FTD n = 128) using *cutpointr*. Area under the curve (AUC) for the receiver-operator characteristic (ROC) and precision-recall (PR) are shown.

Dataset	N	N_Features	Accuracy	Sensitivity	Specificity	F1	AUC-PR	Train Test Gap
**CV (5-fold)**	172	44	NA	NA	NA	NA	0.53	0.17
**Training**	172	44	0.76	0.82	0.74	0.67	0.83	NA
**Test**	57	44	0.75	0.67	0.81	0.67	0.75	0.08

Supplementary Table 4. Performance metrics of the XGBoost model for ALS/FTD spectrum classification.

The model was trained on 172 samples and validated on 57 test samples using 44 features. Metrics include accuracy, sensitivity, specificity, F1 score, and AUC-PR. Cross-validation (CV) results are shown using 5-fold validation. Train-test gap indicates the difference between training and test set AUC-PR.

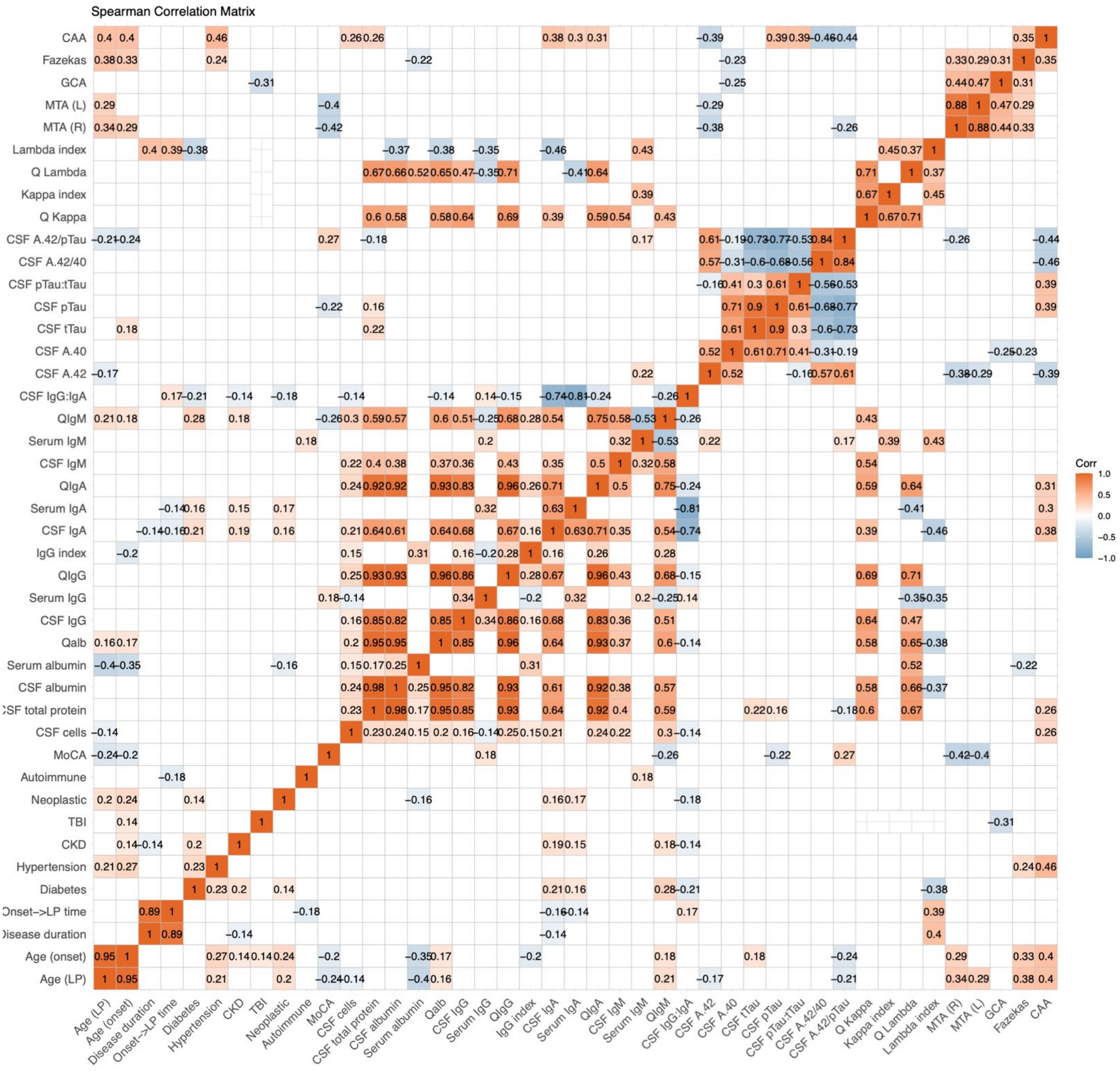

Supplementary Figure 1. Feature correlation matrix

A) Spearman correlations between included numeric features (number inset). Insignificant features (p<0.05) left blank.

A

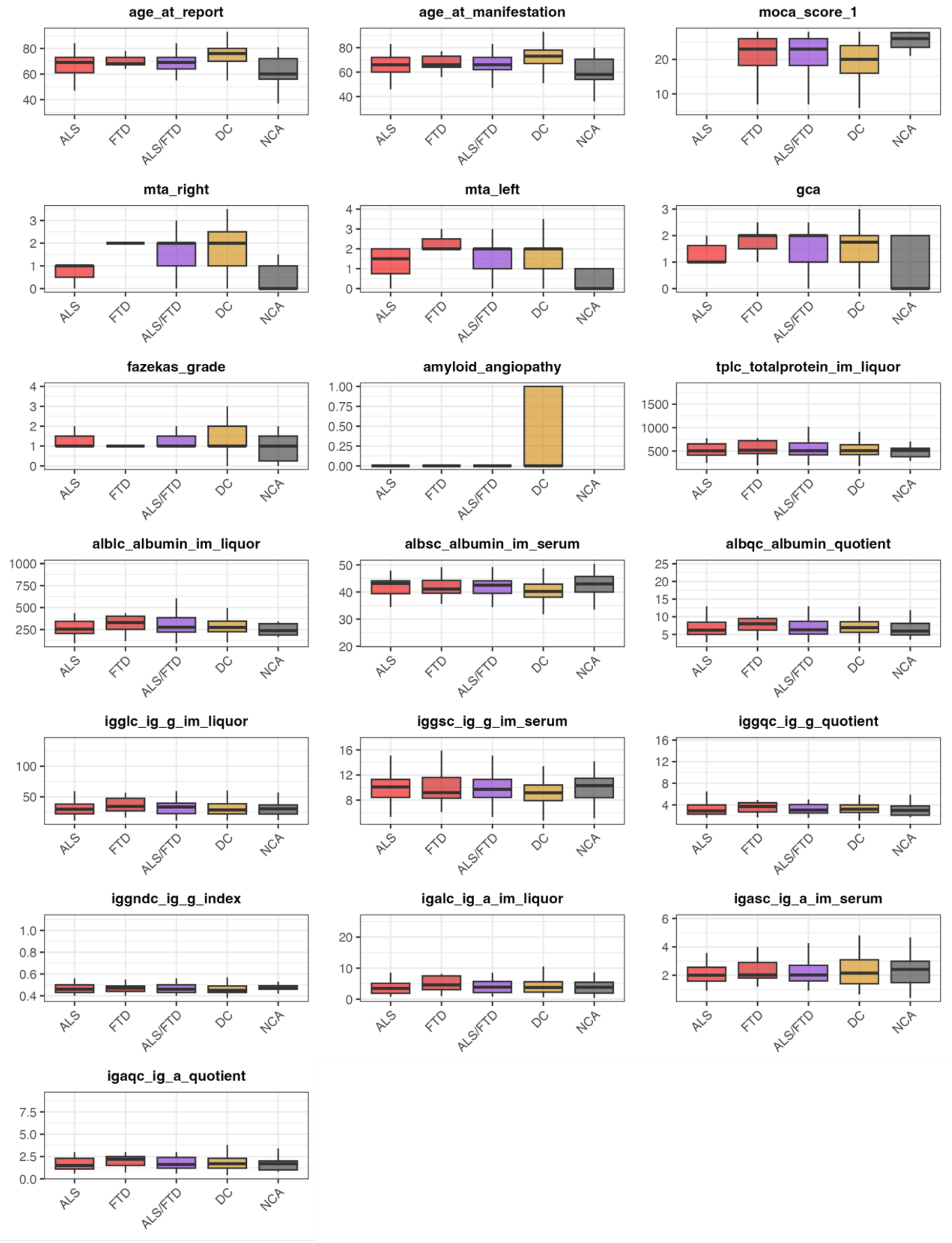
age_at_report
age_at_manifestation
moca_score_1
mta_right
mta_left
gca
fazekas_grade
amyloid_angiopathy
tplc_totalprotein_im_liquor
alblc_albumin_im_liquor
albsc_albumin_im_serum
albqc_albumin_quotient
igglc_ig_g_im_liquor
iggsc_ig_g_im_serum
iggqc_ig_g_quotient
iggndc_ig_g_index
igalc_ig_a_im_liquor
igasc_ig_a_im_serum
igaqc_ig_a_quotient
ALS
FTD
ALS/FTD
DC
NCA

B

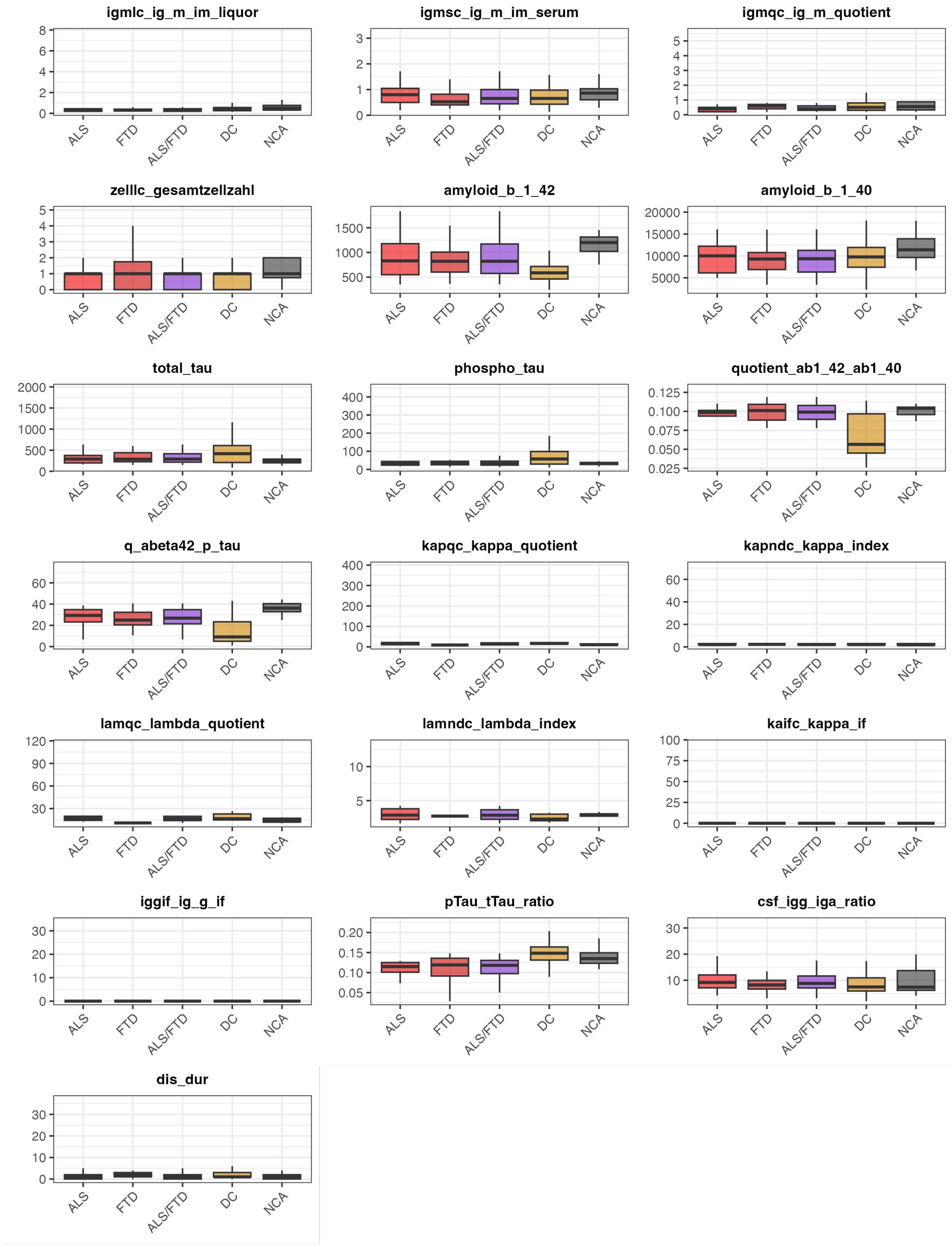

<u>Supplementary Figure 2. Comparison of ALS, FTD and ALS/FTD biomarker profiles.</u>

A-B) Unweighted distributions of 38 CSF, clinicodemographic and MRI features across 5 diagnostic groups. Boxplots illustrate raw biomarker distributions across ALS, FTD, ALS/FTD (i.e., union of ALS and FTD cases), disease controls (AD, 4R_Tau, LBD and NPH) and normal cognitive aging (NCA).

A

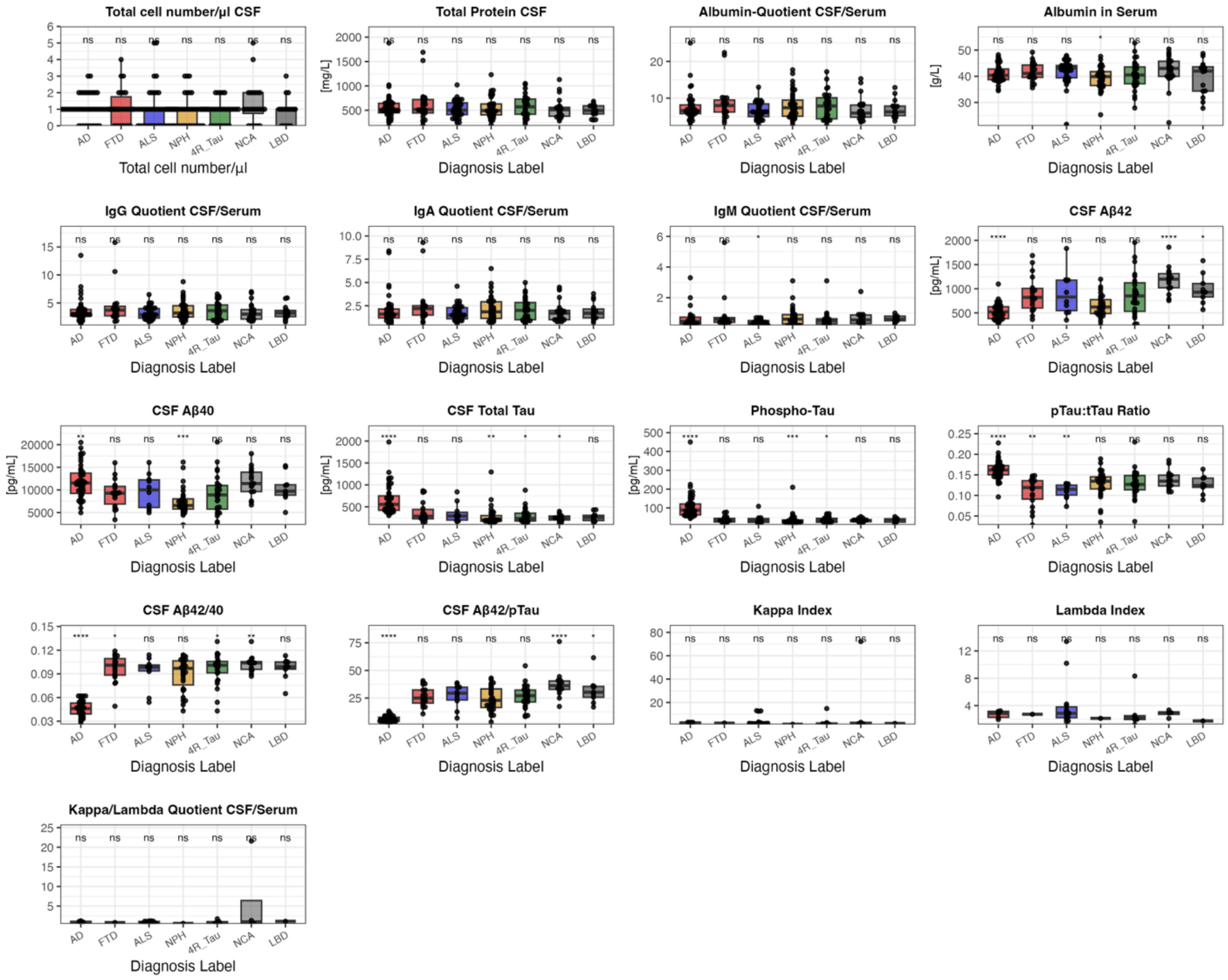

Total cell number/µl CSF
Total cell number/µl
Total Protein CSF
[mg/L]
Albumin-Quotient CSF/Serum
Albumin in Serum
[g/L]
IgG Quotient CSF/Serum
IgA Quotient CSF/Serum
IgM Quotient CSF/Serum
CSF Aβ42
CSF Aβ40
CSF Total Tau
Phospho-Tau
pTau:tTau Ratio
CSF Aβ42/40
CSF Aβ42/pTau
Kappa Index
Lambda Index
Kappa/Lambda Quotient CSF/Serum
[pg/mL]
Diagnosis Label
AD
FTD
ALS
NPH
4R_Tau
NCA
LBD

B

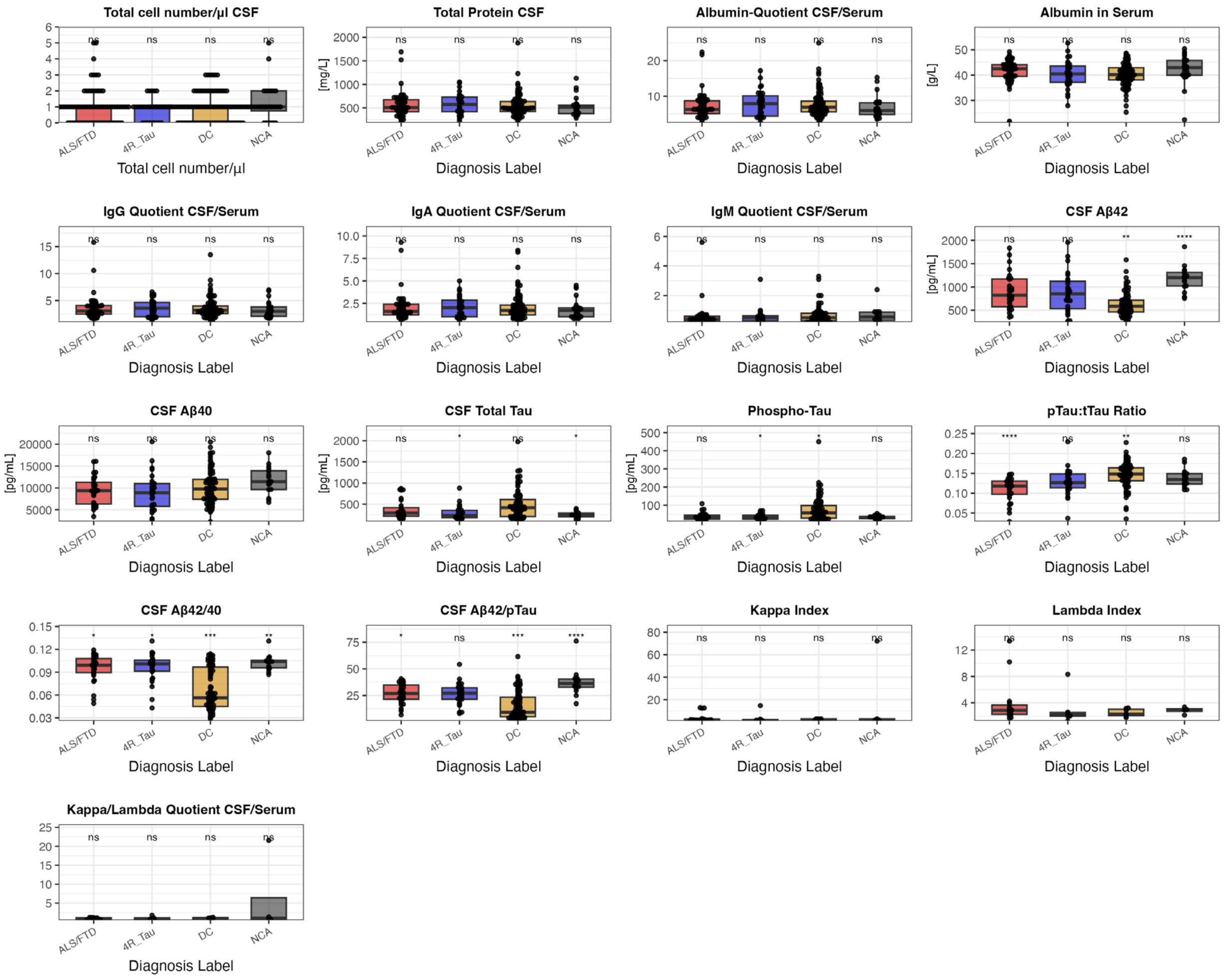

<u>**Supplementary Figure 3. Unweighted biomarker distributions across diagnostic categories**</u>

A) Unweighted distributions of 17 CSF biomarkers across seven diagnostic categories. Box-and-jitter plots illustrate raw biomarker distributions across ALS, FTD, 4R_Tau, AD, LBD, NPH and NCA. Asterisks indicate one-vs-all statistical comparisons using Wilcoxon tests; *p<0.05

B) Unweighted distributions of 17 CSF biomarkers across collapsed four diagnostic groups. Box-and-jitter plots show raw biomarker distributions across ALS/FTD, 4R_Tau, Disease Control (AD, LBD, NPH) and NCA. Asterisks indicate one-vs-all statistical comparisons using Wilcoxon tests; *p<0.05**.**

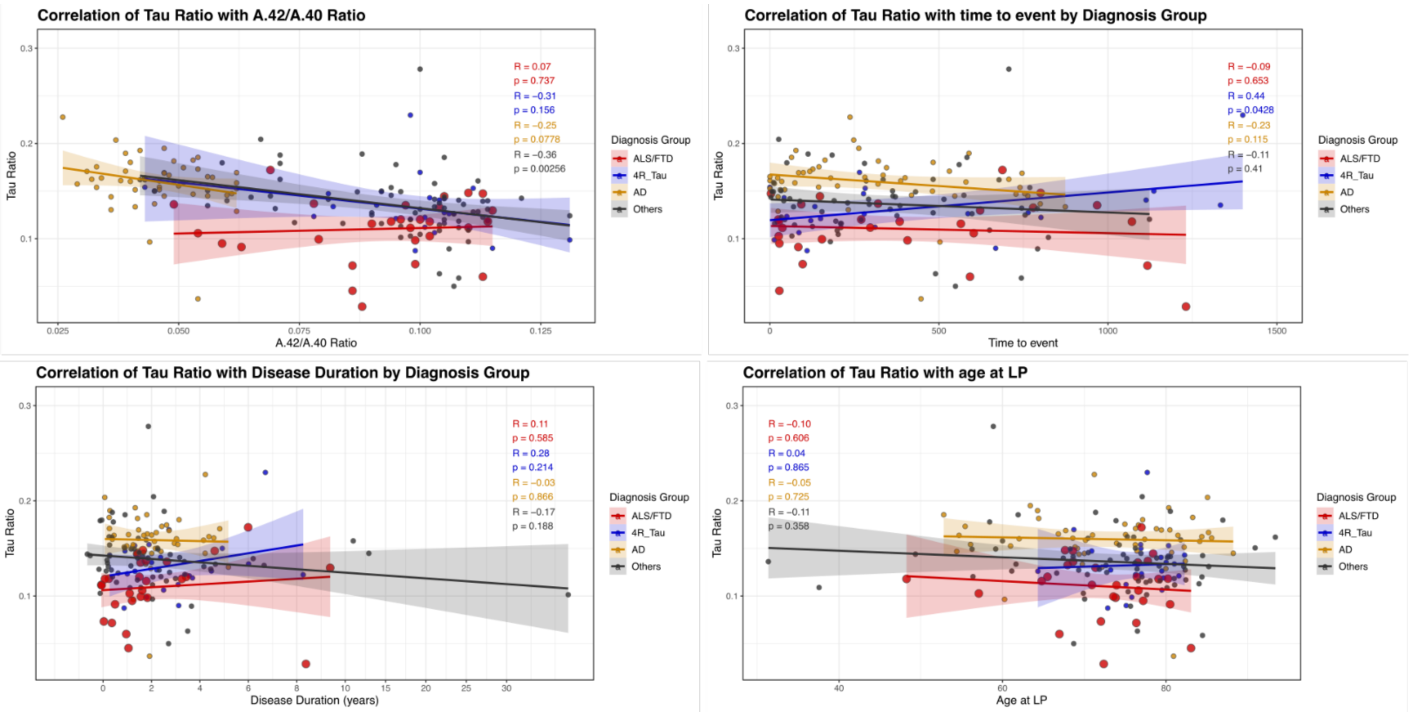

Supplementary Figure 4. Correlations of pTau:tTau ratio with clinical parameters and biomarkers

Scatterplots illustrating the correlation of the pTau:tTau ratio with various clinical parameters and biomarkers. Values indicate results from Spearman correlation, differentiated by diagnosis groups.

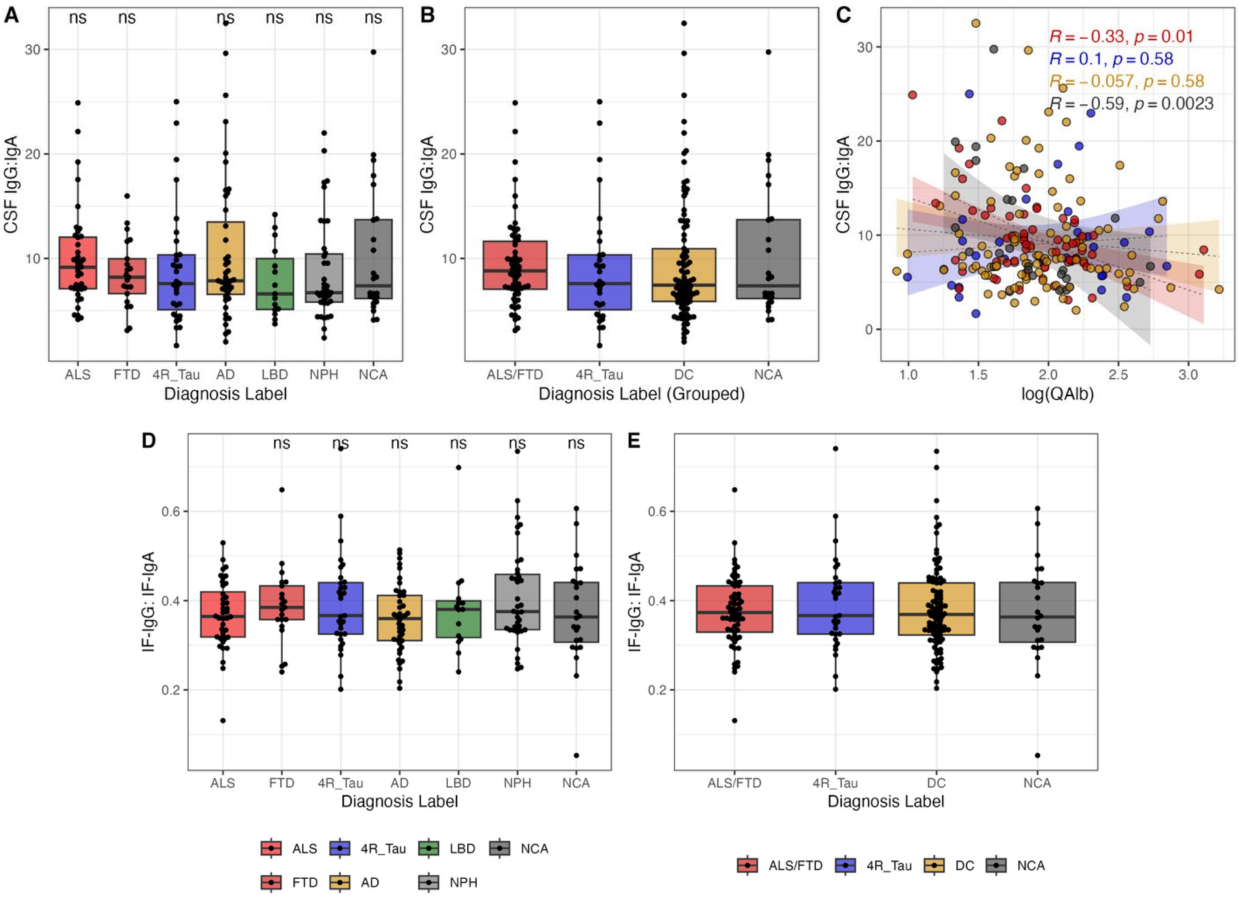

Supplementary Figure 5. The CSF IgG:IgA ratio across neurodegenerative diagnoses

(**A**) Between-group comparisons of CSF IgG:IgA ratio among 4R_Tau vs. other diagnostic categories were assessed using Wilcoxon tests. ns - not significant.
(**B**) Between-group differences in CSF IgG:IgA ratio across grouped diagnoses were assessed using Wilcoxon test. ns - not significant.
(**C**) Scatterplot of the Spearman correlations between CSF IgG:IgA ratio and QAlb. X-axis is log-scaled, with separate linear fits and confidence intervals for diagnosis groups.
(**D**) Between-group comparisons of the ratio of intrathecal fractions (IF) of IgG and IgA among 4R_Tau vs. other diagnostic categories were assessed using Wilcoxon tests. ns - not significant.
(**E**) Between-group differences in IF-IgG:IF-IgA ratio across grouped diagnoses were assessed using Wilcoxon test. ns - not significant.
